# Supplementary material for: Pooled clone collections by multiplexed CRISPR-Cas12a-assisted gene tagging in yeast
Source: Nat Commun. 2019 Jul 4;10:2960. doi: 10.1038/s41467-019-10816-7 (PMC6609715; doi:10.1038/s41467-019-10816-7)
Supplement: Supplementary file 1 — Supplementary Information [file 41467_2019_10816_MOESM1_ESM.pdf]

SUPPLEMENTARY INFORMATION

for

Buchmuller and Herbst *et al.*

Pooled clone collections by multiplexed CRISPR-Cas12a-assisted gene tagging in yeast

CONTENTS

Supplementary Notes

Supplementary Figures

Supplementary Tables

Supplementary Data (Oligonucleotide sequences)

Supplementary References

SUPPLEMENTARY NOTES

Supplementary Note 1. Design of an oligonucleotide pool for C-terminal tagging of yeast ORFs.

The design principles described below are implemented in *R* (https://github.com/knoplab/castR/tree/v1.0). For oligonucleotide design the annotated yeast reference genome was retrieved from the *Saccharomyces* Genome Database[1] (www.yeastgenome.org, November 2016), which contained 5,743 chromosomal yeast ORFs. This process consists of three stages. In stage 1 the CRISPR target sequences are retrieved and evaluated; in stage 2 the corresponding oligonucleotides are constructed *in silico* and evaluated; and in stage 3 the selection of oligonucleotides is further confined to optimally use pool synthesis capacity.

Stage 1. For C-terminal tagging, all protospacer-adjacent motifs (PAMs) of the selected CRISPR-Cas12a endonuclease within ±30 nt around the STOP codon were retrieved. For tagging with FnCas12a, these PAMs were TTV and TYN in the first genome-wide pool design and TTV in the second genome-wide pool design. If a PAM was followed by another PAM, only the downstream PAM was considered to obtain non-adjacent CRISPR target sequences ('spacers') only. In the next step, the 20 nt long target sequences were extracted. Target sequences that contained runs of more than five thymidine bases were removed to avoid pre-mature termination of crRNA transcription by RNA polymerase III. Spacers pre-maturely abrogating transcription by ending with T or TT were not removed because 18 nt spacers can still be functional[2].

For the remaining spacers, we established (1) whether the genomic target sequence would be removed by SIC integration, i.e. whether it spanned the STOP codon, and (2) the risk of off-target cleavage. This risk was assessed as the number of genome-wide occurrences of these sequences (including PAM-free sites) permitting mismatches in the spacer according to the following rules: maximum 1 base mismatch in the crRNA seed, i.e. the first eight PAM-proximal nucleotides, and/or maximum 3 mismatched bases in the entire target sequence. In total, 37,438 TTV sites and 45,445 TYN sites were evaluated during the first genome-wide pool design.

Stage 2. Oligonucleotides are constructed as outlined in Supplementary Figure 6. In brief, the length of each homology arm is established first. Any bases potentially removed from the open reading frame (ORF) or the 3' untranslated region (3' UTR) after target cleavage by the CRISPR endonuclease are reconstituted by the homology arms after SIC integration.

Next, the oligonucleotides are assembled starting with the last ~30 nt of the crRNA expression unit, i.e. of the *SNR52* promoter (first genome-wide pool design) or the tG(GC)F2 tRNA site (second genome-wide pool design). In both designs, the next element is the direct repeat sequence of FnCas12a and the target-specific spacer sequence determined in stage 1. Following, the 3' homology arm, a restriction site used in the recombineering procedure (*Bst*XI in both designs), the 5' homology arm (without the STOP codon) and finally an in-frame linker, such as a Thr-Ser linker or a S1/S3 linker. The linker serves as generic recombineering site with the feature cassette encoding the desired reporter gene.

After *in silico* design, oligonucleotide sequences that contain more than one of the endonuclease restriction sites are removed. Therefore, it is advised to choose a restriction enzyme with low restriction site frequency at the desired tagging site. For C-terminal tagging in yeast, we considered *Bst*XI or *Ngo*MIV in the first pool design (16,005 remaining TTV candidates; 18,720 remaining TYN candidates) and *Bst*XI in the second pool design (32,602 remaining TTV candidates).

Stage 3. Since the number of candidate oligonucleotide designs typically exceeds the synthesis capacity for a single array-based oligonucleotide pool, a selection procedure for pool assembly is required.

In the first genome-wide pool design, this was effected as follows: All ORFs that were targeted by a single oligonucleotide only were preserved regardless of the following criteria. For all other ORFs, oligonucleotides that contained a spacer with low off-target estimate and/or the ability to remove the genomic target by SIC integration were preferred. The final pool contained 12,472 oligonucleotide sequences for tagging 5,664 ORFs.

In the second genome-wide pool design, we tried to consider the parameters previously identified to improve tagging success. Therefore, oligonucleotide candidates were ranked based on a bonus-system. For each ORF, the three oligonucleotides with the highest bonus were selected (17,691 oligonucleotides). We also included designs that had proven repeatedly successful in the first genome-wide pool design, summing up to 18,752 sequences. Another 8,248 candidate oligonucleotides were included by random draw to use up the remaining synthesis capacity of this pool (27,000 oligonucleotides) for tagging 5,940 ORFs.

Supplementary Note 2. CASTLING and gene drive

Circumstances under which CASTLING can generate a gene drive-system.

The co-occurrence of a gene with a molecular machinery that is suited to propagate itself along with that gene from one chromosome to a homologous one, leading to

homozygotization of the gene locus with the propagation machinery biases the inheritance of this gene within sexually reproducing populations towards much higher transmission frequencies than expected from sexual inheritance (‘gene drive’). Importantly, the strength of the gene drive depends primarily on the fidelity and efficiency by which the element is copied, and less on the nature of the gene that is driven. In each generation, genes that decrease the fitness of an individual can spread just as efficiently as genes that confer e.g. resistance to antibiotics, leading to potentially harmful or undesired effects on the host population (‘population replacement’[3,4], spread of resistances[5]).

Artificial genetic elements that combine the expression of an RNA-programmable endonuclease (i.e. Cas9 or Cas12a) and a gene from which a cognate crRNA is expressed can exhibit gene drive in a process reminiscent of gene conversion[6].

CASTLING bears the risk of accidentally crating such an artificial drive system if one of the ‘self-integrating’ cassettes is targeted to the neighborhood of a stably integrated, actively expressed Cas12a-endocing gene (Supplementary Figure 11). In such a constellation, the crRNA expressed from the gene engineered by CASTLING could cleave the homologous chromosome and thereby induce repair of the damaged locus with the information of the engineered chromosome. If the CRISPR endonuclease-encoding locus is copied along with the repair template during this process, homozygotization of the drive system is complete. Thus, the likeliness and the strength of the drive depends on the distance between the Cas12a and the crRNA gene, i.e. on the amount of sequence between them that is homologous to the sequence on the homologous chromosome. The exact distance that will eliminate gene drive capabilities is not known, but given the fact that already 36 bp in *S. cerevisiae* (which may be different for other hosts) are sufficient to promote homologous recombination efficiently, we expect that the ability to ‘drive’ will be lost or strongly reduced if the Cas12a gene and the crRNA gene are separated by more than a few hundred base pairs of sequence that is homologous to the other chromosome.

Safety and security measures.

Based on these considerations we strongly recommend the following safety measures that are effective to prevent the accidental creation of an artificial genetic element that exhibits gene drive using CASTLING in sexually reproducing organisms:

- The Cas12a endonuclease can be contained on an episomal plasmid that is not prone to integrate into the genome. Plasmids that contain counter-selectable markers such as *URA3* can be used to enforce the loss of the Cas12a after library preparation.
- In case a strain with a genomically integrated Cas12a gene is used, the SICs should not target sequences in proximity to the Cas12a locus. In this situation, occasional spontaneous integration of the plasmids into the genome must be considered and care must be taken that none of the SICs can target the plasmid itself. Creating such crRNAs can be avoided by removing (or masking) all sequences that fulfill these criteria from the host reference genome sequence before starting with oligonucleotide pool design.

If these minimal requirements are met and if all the generated genetically modified organisms (GMOs) are contained properly during the experiment, we believe that CASTLING bears no risk of gene drive creation.

SUPPLEMENTARY FIGURES

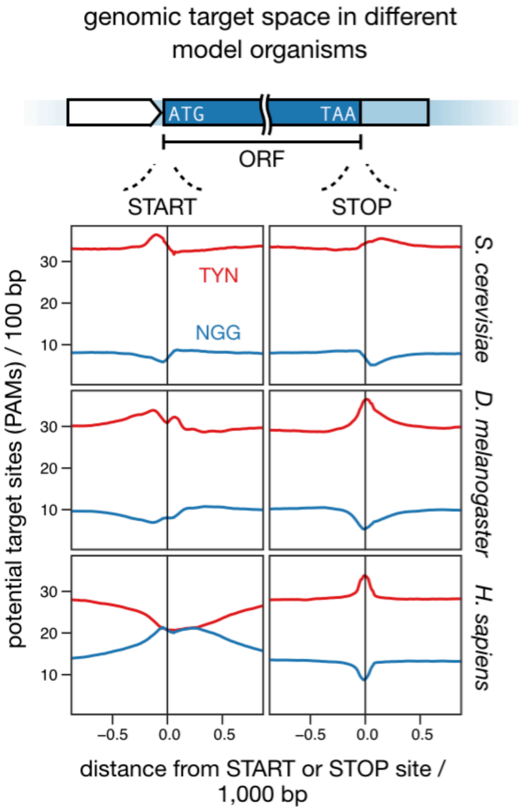

Supplementary Figure 1. Genomic target space in different organisms considering PAM sites from Cas9 and Cas12a endonucleases.

The frequency of the protospacer-adjacent motifs (PAMs) for CRISPR-Cas9 (NGG) and CRISPR-Cas12a (TYN) endonucleases per 100 bp (sliding-window, increment 10 bp) ±1 kb around the START (ATG) and STOP codon (e.g. TAA) of open-reading frames (ORFs) in different organisms is shown. As previously reported[7], GC-content usually drops or peaks at such sites.

Source data are provided as a Source Data file.

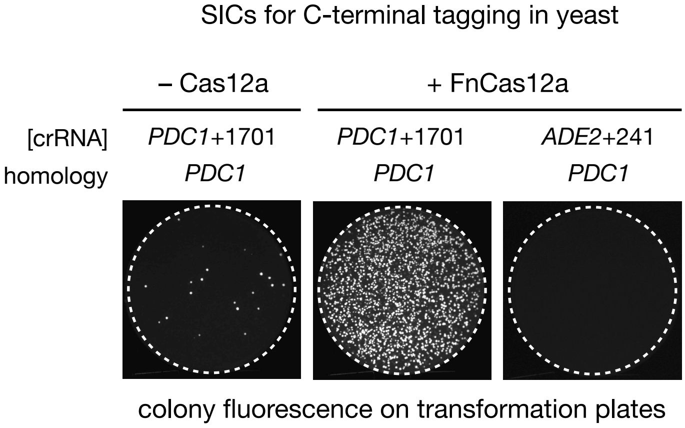

Supplementary Figure 2. Specific and efficient SIC integration in CASTLING.

Transformation of a SIC for C-terminal tagging of *PDC1* using 50 nt homology arms, with and without expression of Cas12a of *Francisella novicida U112* (FnCas12a), or with a crRNA targeting *PDC1* at base +1701, or the *ADE2* locus at base +241 (*ade2* knock-out observed).

Source data are provided as a Source Data file.

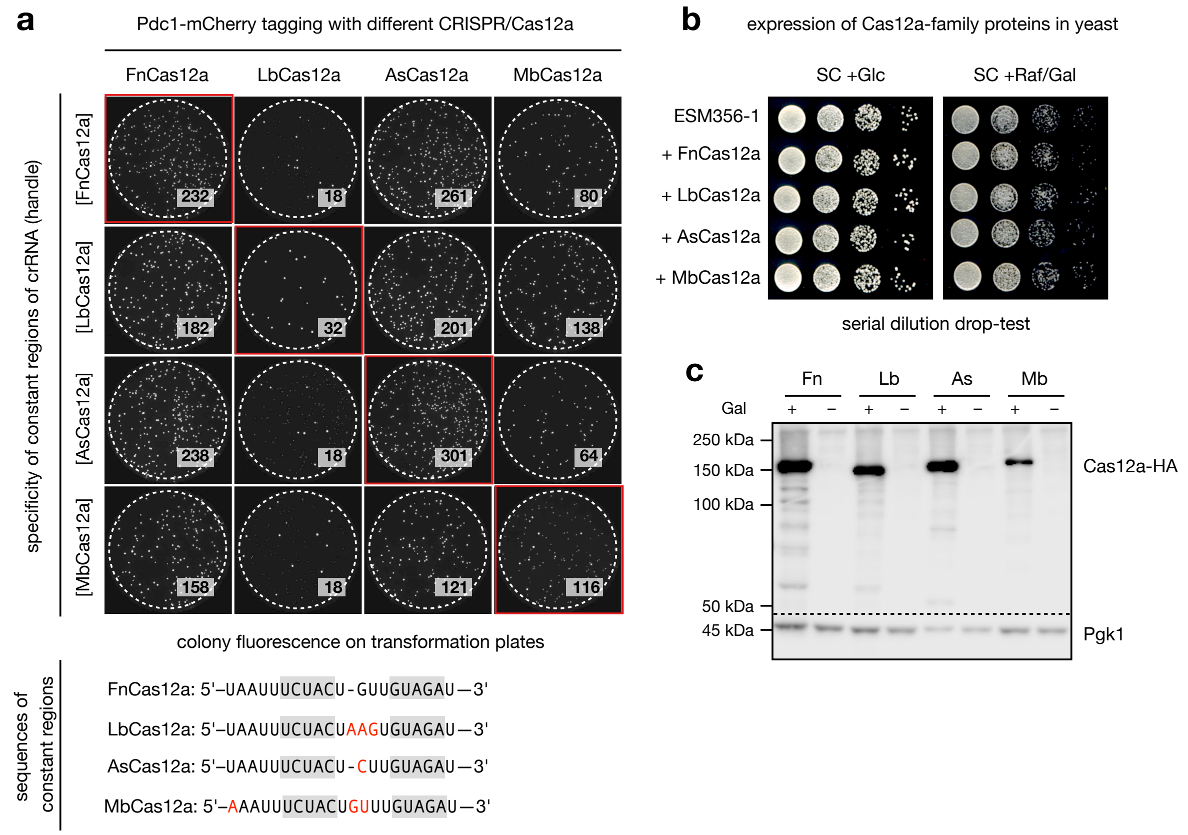

Supplementary Figure 3. Evaluation of Cas12a-family proteins and expression systems for gene tagging using 'self-integrating cassettes' in yeast.

(**a**) Combinatorial testing of four different CRISPR-Cas12a-family proteins and four different constant regions ('handles') of the crRNA for tagging *PDC1* with a red fluorescent reporter protein (mCherry) by means of [crRNA]*PDC1*+1701 (same PAM, TTTA, in all cases). Colony fluorescence is shown after spread-plating the transformation reaction and outgrowth; numbers indicate the fluorescent colonies growing on the plates after replica-plating. The results demonstrate that integration efficiency of SICs is less dependent on the handle than on the identity of the Cas12a protein.

(**b**) Serial dilutions of yeast cultures to test potential toxicity of different CRISPR-Cas12a-family proteins[2] under expression from a glucose-repressed/galactose-inducible promoter, namely Cas12a of *Francisella novicida U112* (FnCas12a), *Lachnospiriceae bacterium ND2006* (LbCas12a), *Acidaminococcus sp. BV3L6* (AsCas12a), and *Moraxella bovoculi 237* (MbCas12a). None of these Cas12a exhibited toxicity in yeast under these conditions.

(**c**) *See page 8.*

(**c**) Expression of all tested Cas12a variants was confirmed by immunoblotting (lower panel). Yeast cell extract was normalized to total cell number 3.5 h post induction with 2% galactose. When grown in 2% raffinose as sole carbon-source (–), Cas12a protein levels were below the limit of detection. The Cas12a-family proteins were detected using a hemagglutinin tag and stained by monoclonal antibodies (12CA5, Sigma-Aldrich). Pgk1 (44.7 kDa) was stained as loading control and detected using monoclonal mouse anti-Pgk1 antibodies (R & D Systems, Fisher Scientific).

Source data of panels a–c are provided as a Source Data file.

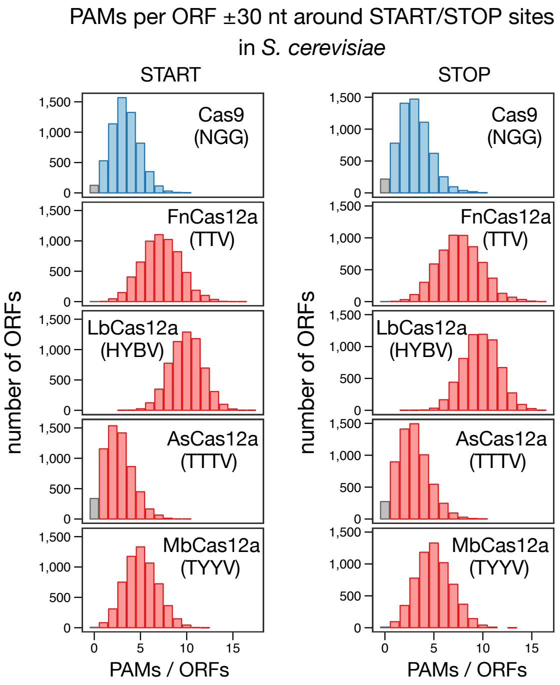

Supplementary Figure 4. Genomic target space of different CRISPR-Cas12a proteins in yeast.

Using CRISPR-Cas12a family proteins, many open-reading frames (ORFs) in yeast can be targeted at multiple sites near (±30 nt) the START or STOP codon. The target space ranges from 83% (AsCas12a) to 99% (FnCas12a) considering all annotated ORFs (retrieved from the *Saccharomyces* Genome Database[1], www.yeastgenome.org, November 2016).

Source data is provided as a Source Data file.

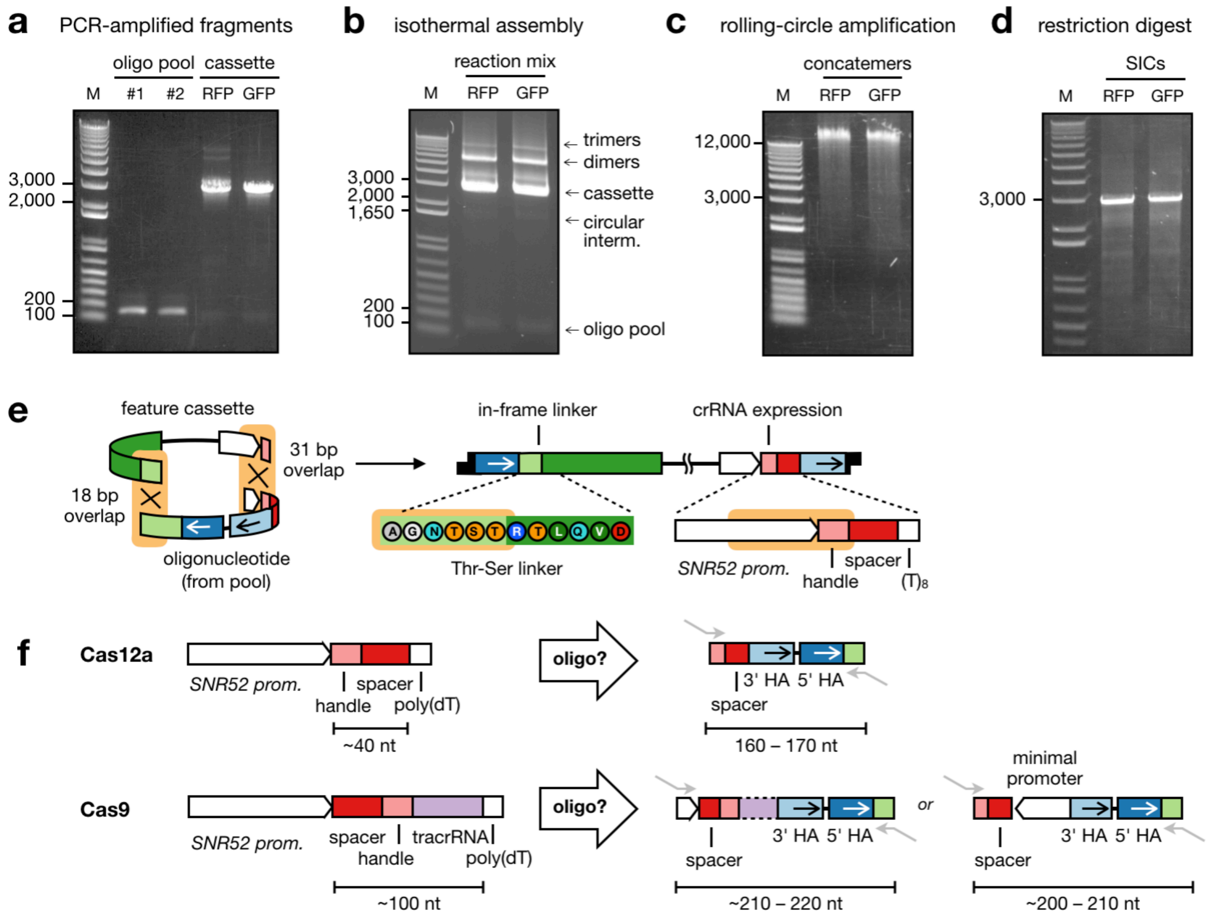

Supplementary Figure 5. Molecular *in vitro* recombineering procedure to create 'self-integrating' cassettes (SICs) from a pool of oligonucleotides. *a–d* displaying agarose gel electrophoresis images of the starting materials, different intermediates and the final SICs.

(**a**) The oligonucleotide pool (< 170 bp) and the desired feature cassettes (here: 2–3 kb, but can be shorter or longer) are PCR-amplified (to introduce short homologous overhangs (15–30 bp) for isothermal assembly).

(**b**) Molecular recombineering by isothermal assembly yields the 'self-integrating cassettes' (SICs) as a covalently-closed circular DNA along with reaction by-products. The circular DNA constitute a minor fraction of the reaction product and for further processing the smallest band is isolated (circular intermediates).

(**c**) The circular DNA serves as a template for random-primed isothermal rolling-circle amplification (RCA) to generate very long (>12 kb) linear dsDNA by means of a highly processive DNA-polymerase with strong strand-displacement capabilities such as phi29 DNA-polymerase. Each linear dsDNA is a concatemer repeating a single SIC.

(**d–f**) *See page 11.*

(**d**) Restriction digestion with a suitable restriction enzyme (here: *Bst*XI) breaks down the long dsDNA molecules into SIC monomers through cleavage of the sequence that linked the homology arms in the designed oligonucleotides.

(**e**) Specification of overlap length for isothermal assembly that collapse into an in-frame peptide linker and a functional gene to drive crRNA expression.

(**f**) Minimal requirements to provide all locus-specific elements on a single oligonucleotide. For Cas12a, the constant crRNA handle (20 nt) can be part of the primer binding site, and thus the total length of the oligonucleotide ranges 160–170 nt. For Cas9, additional elements must be provided inside the oligo, either a tracrRNA (60 nt) that can be fused with the crRNA to yield a sgRNA (90 nt) or – to reverse the orientation of the elements – a minimal constitutive RNA-polymerase III promoter (35 nt). In total, such oligonucleotides would range 200–220 nt in length. Given current limitations in oligonucleotide synthesis, shorter oligonucleotides (i.e. the Cas12a design) are preferred.

Source data of panels a–d are provided as a Source Data file.

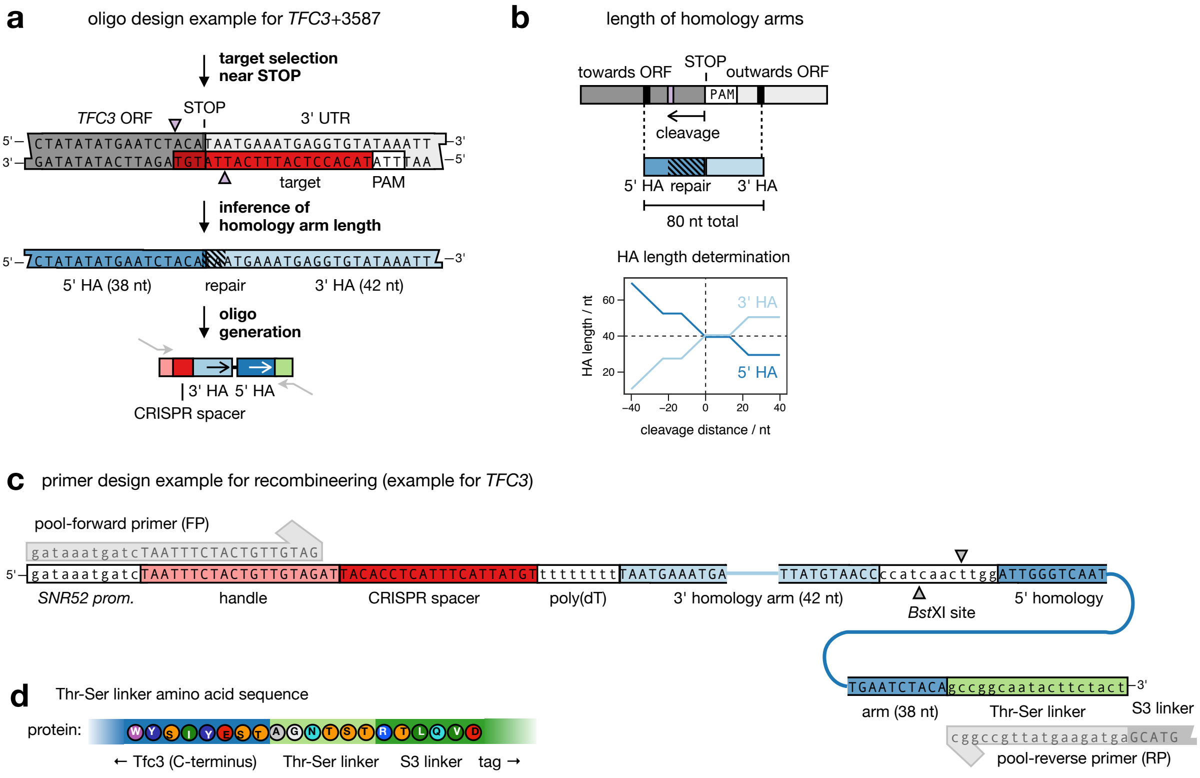

Supplementary Figure 6. Design example for an oligonucleotide to create a SIC for tagging the gene *TFC3* at position +3587, near the STOP codon.

(**a**) Locus-specific elements that are included in the oligonucleotide are the CRISPR spacer complementary to the target sequence for CRISPR-Cas12a, and the homology arms that precisely end and start with the STOP codon for C-terminal tagging.

(**b**) The length of homology arms is chosen to always sum up to a total of 80 nt (for space constraints on the oligonucleotide). If the DNA double-strand break occurs upstream of the STOP codon, a part of the 5’ homology arm (HA) serves to reconstitute the lost sequence information and is therefore chosen longer. Experimentally, we determined that homology arms as short as 20 nt still function efficiently to guide correct insertion of the SICs (data not shown).

(**c**) Primer design example (as used for the libraries in Figure 4, ID #1.1–1.3 in Supplementary Table 2) for the forward (FP) and reverse primer (RP) of pool amplification. The FP overlaps with the *SNR52* promoter that drives crRNA expression, the RP translates in an in-frame linker between the ORF and the desired tag.

(**d**) In-frame peptide linker after C-terminal tagging of Tfc3.

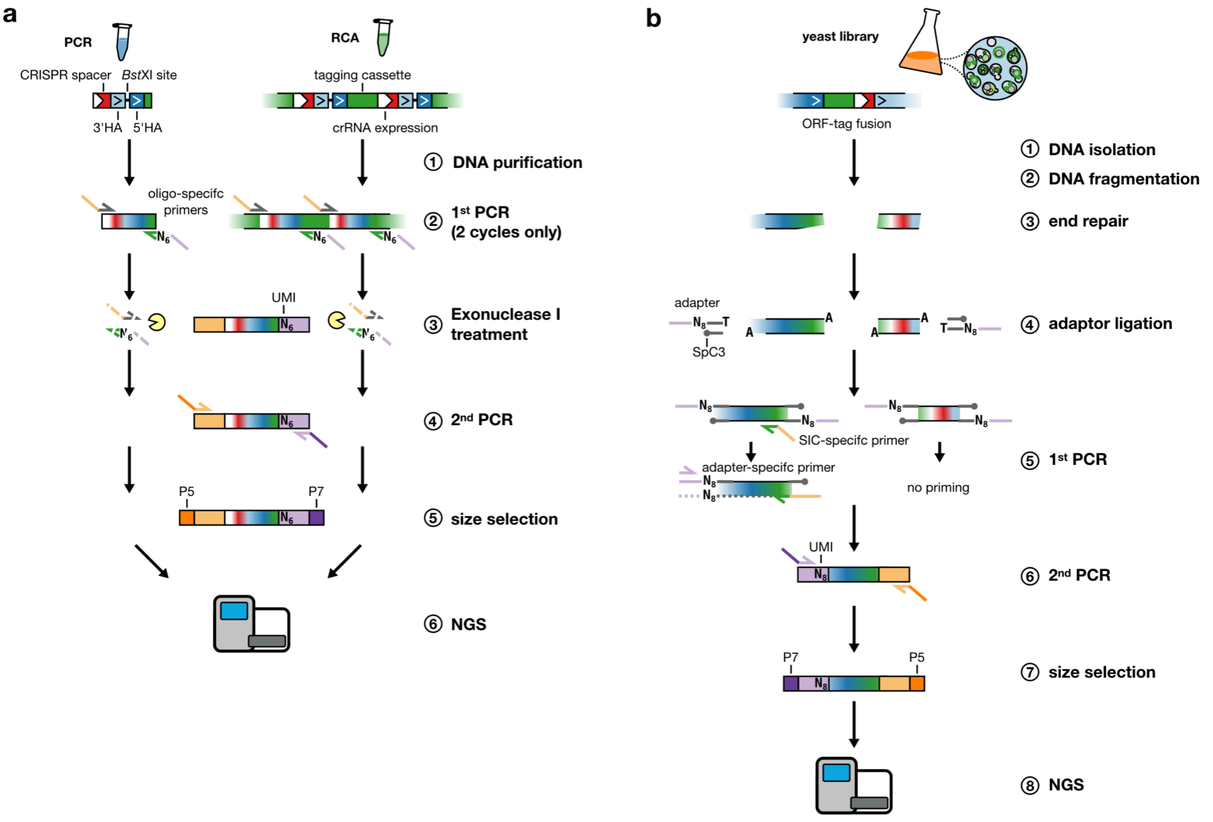

Supplementary Figure 7. Next-generation sequencing (NGS) library preparation with unique molecular identifiers (UMIs) for molecule counting.

(**a**) Quantitative NGS of molecular recombineering intermediates used for CASTLING. The PCR-amplified oligonucleotide pools (left side) or the undigested (concatemeric) SICs yielded by rolling circle amplification (RCA, right side) are quantitated using a random hexameric oligonucleotide sequence (N_6) as UMI. This N_6 UMI is incorporated in two rounds of primer annealing and elongation (1st PCR). Unincorporated primers are then destroyed by Exonuclease I treatment, followed by heat inactivation of Exonuclease I. In a final PCR (30 cycles), technical sequences required for Illumina NGS such as P5 and P7 along with barcodes to distinguish different samples are attached.

(**b**) For quantitative Anchor-Seq of the yeast libraries, adapters with random octameric nucleotides (N_8) are ligated to the fragmented and end-repaired genomic DNA. Next, the fragments which contain SIC-derived sequences (the figure shows the junction of the ORF and the tag) are selectively amplified in a PCR with a SIC-specific and a reverse primer. During the initial cycle, the SIC-specific primer generates the binding site of the reverse primer by replication of an asymmetric sequence. This allows for these genomic fragments to be exponentially amplified. An elongation-inhibiting group (Spacer C3, SpC3) on the complementary strand of the ligated adapters prohibits non-specific amplification of fragments without the SIC-specific sequence of interest.

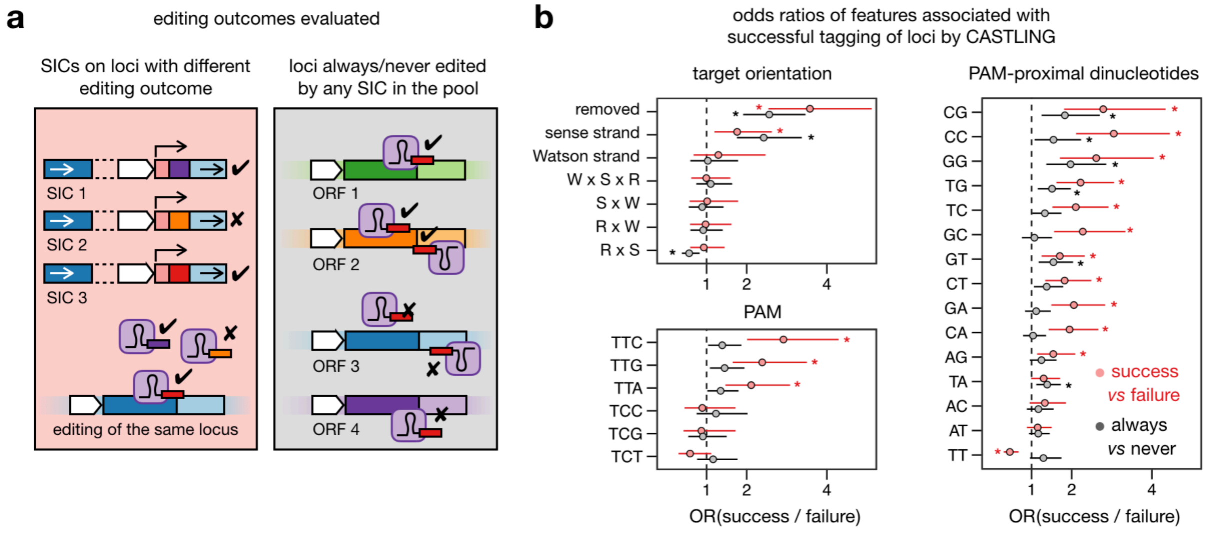

Supplementary Figure 8. Features of the crRNA and target locus associated with successful tagging.

(**a**) Details to the analysis shown in Figure 4d. Two scenarios were considered as represented by red and grey panels. Scenario 1 (red panel) considers all SICs demonstrably present in the SIC pool for the same gene to be tagged, comparing them based on the editing outcome (success: *N* = 2,053 SICs; failure: *N* = 2,488 SICs). Only genes were included in this analysis, that had both, successful and non-successful SICs. Scenario 2 (gray panel) compares tagging success for genes, in which any of the designed SICs promoted (total *N* = 2,885 SICs) or failed (*N* = 3,209 SICs) to tag the gene.

(**b**) Odds ratio (OR) based on samples drawn akin to (*a*) based on multiple logistic regression analysis. Asterisks indicate properties for which the impact on odds is significantly (Fisher's exact test, $p < 0.01$) different from 1 (no effect); error bars represent 95% confidence intervals). All scenarios displayed were checked for inter-dependencies (indicated as 'A x B') also in-between the panels with no effect on the general outcome, but were split for the sake of clarity. 'Sense strand' refers to the genomic target occurring on the non-transcribed strand, whereas 'Watson strand' is any of the two strands as specified in the yeast gene nomenclature. 'Nucleosome occupancy at the PAM' or across the entire 'target sequence'[8] and 'gene expression in YP-Raf/Gal' (medium to transiently express FnCas12a) or 'YPD'[9] (to suppress FnCas12a expression) were also analyzed with no significant impact on tagging success (data not shown).

Source data are provided as a Source Data file.

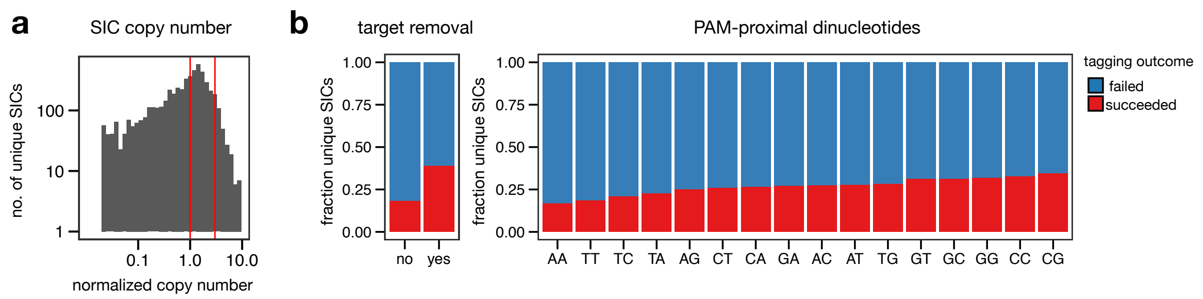

Supplementary Figure 9. Verification of features of the crRNA and the target locus that are associated with successful tagging.

(**a**) Copy number distribution of the SICs in the SIC pool after RCA. Integration success of a particular SIC is likely to be influenced by its copy number in the SIC pool.

(**b**) To validate the impact of different features on the tagging success, we restricted the analysis of the impact of different features on tagging success to SICs with similar representation in the SIC pool (1- to 3-fold of the median copy number, region marked with red lines in (a)). Target removal (left panel): Comparison of SICs on the basis whether their integration does remove the target site or not. Influence of PAM-proximal dinucleotides: Comparison of the influence of the PAM-proximal dinucleotides in the crRNA sequence on tagging success (right panel). Both plots demonstrate that tagging success was higher when the rule set (Supplementary Figure 8) was obeyed.

Source data are provided as a Source Data file.

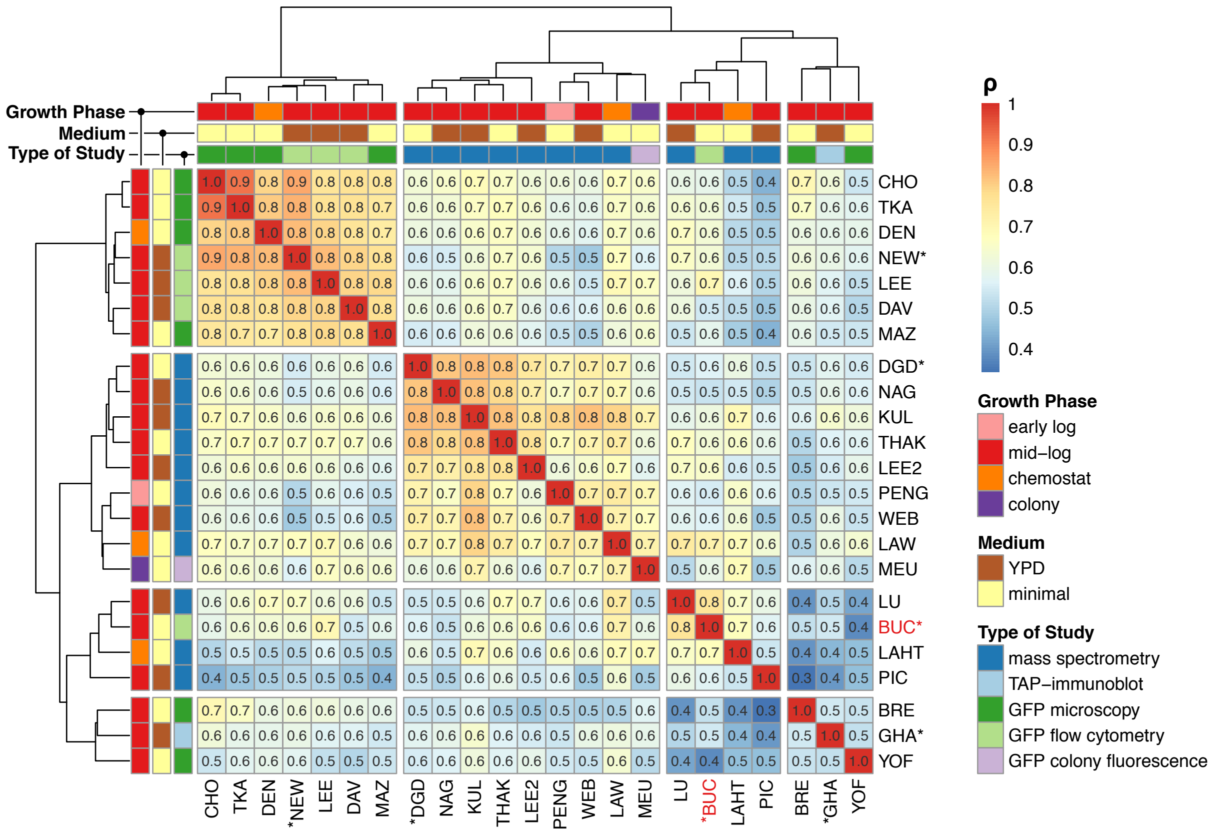

Supplementary Figure 10. Spearman correlation coefficients (ρ) between protein abundance estimated after phenotyping by FACS in this study (BUC, marked red, Figure 5d–f) and other studies. Study abbreviations as in Ref[10] except for (MEU)[11] and this study (BUC). An asterisk indicates studies that appear in Figure 5e. Additional information about each study (growth phase, medium, type of study) were compiled from information in Ref[10].

Source data are provided as a Source Data file.

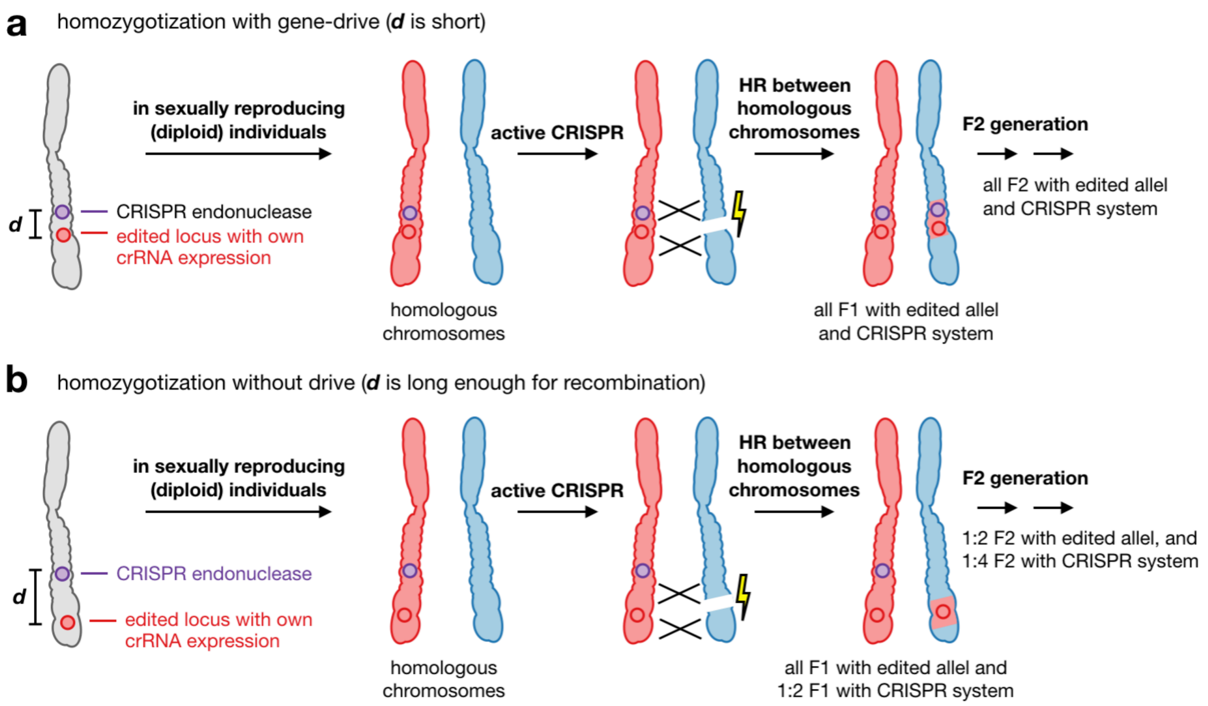

Supplementary Figure 11. Mechanism of allele homozygotization and potential 'gene drive' with stably integrated CRISPR endonucleases (Supplementary Note 1).

(**a**) If the distance (***d***) of the crRNA expression site and the endonuclease locus is very short, homozygotization of both loci is highly likely (gene drive).

(**b**) With increasing ***d***, the likelihood that both genes are copied to the homologous chromosome decreases.

SUPPLEMENTARY TABLES

Supplementary Table 1. Library statistics for libraries created from pools A, B1 and B2 (Figure 3).

ID	oligo-nucleotide pool	PCR pool (after amplification)			feature cassette	SIC pool (after recombineering/RCA)			clone collections (libraries)			
		primer	reads mapped	identified unique sequences, ORFs (UMI >2)		pooled RCA reactions	reads mapped	identified unique sequences, ORFs (UMI > 2)	clones	library stock	reads mapped	identified unique sequences, ORFs (UMI >2)
1a	pool A, 40.0 ng (0.8 pmol)	pool-FP3 + pool-RP3	152,011	1,200 seq. (76%), 206 ORFs (96%)	pKH51	2	125,015	493 seq. (31%), 147 ORFs (68%)	58,000	YKH231	277,026	280 seq. (18%), 125 ORFs (58%)
1b			195,535	1,281 seq. (81%), 209 ORFs (97%)	pKH51	2	70,984	324 seq. (20%), 116 ORFs (54%)	64,000	YKH232	266,519	202 seq. (13%), 107 ORFs (50%)
2a	pool B1, 150. pg (3.0 fmol)	pool-FP2 + pool-RP2	265,556	1,308 seq. (83%), 209 ORFs (97%)	pKH51	3	177,648	872 seq. (55%), 191 ORFs (89%)	82,000	YKH227	443,204	496 seq. (31%), 172 ORFs (80%)
2b			253,732	1,333 seq. (84%), 209 ORFs (97%)	pKH51	2	225,114	601 seq. (38%), 179 ORFs (83%)	81,500	YKH228	478,846	309 seq. (19%), 144 ORFs (67%)
3a	pool B2, 150. pg (3.0 fmol)	pool-FP3 + pool-RP3	148,529	1,454 seq. (92%), 211 ORFs (98%)	pKH51	2	44,005	825 seq. (52%), 177 ORFs (82%)	58,500	YKH229	192,534	233 seq. (15%), 127 ORFs (59%)
3b			143,308	1,435 seq. (91%), 211 ORFs (98%)	pKH51	2	133,050	1,082 seq. (67%), 196 ORFs (91%)	32,000	YKH230	276,358	265 seq. (17%), 135 ORFs (63%)
4a	pool B2, 1.0 ng (20 fmol)	pool-FP3 + pool-RP3	179,709	1,399 seq. (89%), 211 ORFs (98%)	pKH51	3	60,323	1,033 seq. (66%), 191 ORFs (89%)	90,500	YKH233	240,032	812 seq. (51%), 197 ORFs (92%)
4b			202,657	1,347 seq. (85%), 211 ORFs (98%)	pKH51	3	52,332	964 seq. (61%), 189 ORFs (98%)	95,000	YKH234	254,989	835 seq. (53%), 197 ORFs (92%)

Supplementary Table 2. Library statistics pool C (Figure 4) and pool D (Figure 5). * = DADA2 de-noising, considering only perfectly matched reads to the reference.

ID	oligo-nucleotide pool	PCR			feature cassette	SIC pool (after recombineering, RCA)			clone collections (libraries)			
		**primer**	**reads mapped**	**identified unique sequences, ORFs**		**pooled RCA reactions**	**reads mapped**	**identified, unique sequences, ORFs**	**clones**	**library stock**	**reads mapped**	**identified unique sequences, ORFs**
#1.1	pool C, 1 x 80 pg (1.5 fmol)	pool-FP1 + pool-RP1	1,610,291	3,802 seq.* (30%), 2,985 ORFs (53%)	pMaM505 (kanMX)	4	3,066,702	3,196 SICs* (26%), 2,522 ORFs (45%)	109,000	YDK553 + YDK557	28,574,949	2,052 ORFs (36%)
#1.2					pBeB019 (hphNT1)	4	n. d.	n. d.	100,000	YDK556 + YDK483	28,619,645	2,317 ORFs (35%)
#1.3	pool C, 10 x 80 pg (1.5 fmol)	pool-FP1 + pool-RP1	18,009,833	8,110 seq.* (65%), 5,025 ORFs (89%)	pBeB019 (hphNT1), 1/10 of PCR	4	n. d.	n d.	76,500	YDK559	22,476,787	2,033 ORFs (36%)
LibA	pool D, 1 x 2 ng (40 fmol)	pool-FP2 + pool-RP2	n.d.	n.d	pBeB064 (kanMX)	30	n.d.	n.d.	704,000	YKG7+ YKG10+ YKG13	52,992,365	3,786 ORFs (64%)
LibB									44,000	YKG5+ YKG8+ YKG11	4,006,055	2,869 ORFs (48%)
LibC					pKH51 (hphNT1)	2	n.d.	n.d.	116,000	YKH225	10,006,829	3,011 ORFs (51%)

Supplementary Table 3. Newly characterized ORFs in terms of protein abundance in liquid minimal medium. The detection probability is a function of the relative abundance of a clone in a sorted pool of cells (or 'bin') and it is influenced by the selectivity of the phenotype enrichment protocol and the accuracy of genotype identification. For each ORF identified after MinION nanopore sequencing, the read count (> 1 count) and enrichment or depletion (column 'fold') in relation to the representation of each ORF in the starting pool, which was characterized using Illumina NGS. Only enriched loci were considered as hits. ORFs for which abundance information was available outside genome-scale experiments[12] are asterisked.

bin	enriched loci			depleted loci		
	systematic name	counts	fold	systematic name	counts	fold
bin 2 (5–24%)				YBR298C-A	5	0.39x
				YJL136W-A	3	0.71x
				YMR175W-A*	7	0.35x
				YOL164W*	3	0.23x
bin 3 (25–44%)	YGR146C-A	412	3.9x	YGR240C-A	8	0.22x
	YGR204C-A	61	2.0x	YJR151W-A	146	0.24x
	YOL156W*	18	4.4x			
	YOR072W-B	2	1.3x			
	YPL038W-A*	17	2.7x			
bin 4 (45–64%)	YDR194W-A*	4	4.5x			
bin 5 (65–89 %)	YGL006W-A	5	10.3x			
	YML054C-A	316	4.8x			
	YMR230W-A*	8	3.1x			
bin 7 (95–99 %)	YBR196C-A*	570	4.2x			

Supplementary Table 4. Strains used in this study.

strain	background	description	purpose	source
**ESM356-1**	S228C	MAT**a** ura3-52 leu2Δ1 his3Δ200 trp1Δ63	auxotrophic laboratory yeast strain	Ref.[13]
**YBeB500**	ESM356-1	*ura3-52*::GAL1pr-FnCas12a::NLS::3xHA-CYC1t (natNT2)	inducible expression in yeast (use discouraged for risk of gene-drive)	this work
**YBeB600**	ESM356-1	*ura3-52*::GAL1pr-LbCas12a::NLS::3xHA-CYC1t (natNT2)	inducible expression in yeast (use discouraged for risk of gene-drive)	this work
**YBeB700**	ESM356-1	*ura3-52*::GAL1pr-AsCas12a::NLS::3xHA-CYC1t (natNT2)	inducible expression in yeast (use discouraged for risk of gene-drive)	this work
**YBeB800**	ESM356-1	*ura3-52*::GAL1pr-MbCas12a::NLS::3xHA-CYC1t (natNT2)	inducible expression in yeast (use discouraged for risk of gene-drive)	this work

Supplementary Table 5. Plasmids used in this study.

plasmid	backbone	description	purpose	source
**pY004**	pcDNA 3.1(+)	CMVpr-hFnCas12a::NLS ::3xHA::bGH poly(A)	mammalian expression	Ref. [2]
**pY010**	pcDNA 3.1(+)	CMVpr-hAsCas12a::NLS ::3xHA::bGH poly(A)	mammalian expression	Ref. [2]
**pY014**	pcDNA 3.1(+)	CMVpr-hMbCas12a::NLS ::3xHA::bGH poly(A)	mammalian expression	Ref. [2]
**pY016**	pcDNA 3.1(+)	CMVpr-hLbCas12a::NLS ::3xHA::bGH poly(A)	mammalian expression	Ref. [2]
**pRS306N**		shuttle vector and yeast integrating plasmid at *ura3-52* conferring nourseothricin resistance		Ref.[14]
**pMaM486**	pRS306N	GAL1pr-hFnCas12a::NLS ::3xHA-CYC1t	stable genomic integration in yeast, inducible expression	this work
**pMaM487**	pRS306N	GAL1pr-hAsCas12a::NLS ::3xHA-CYC1t	stable genomic integration in yeast, inducible expression	this work
**pMaM488**	pRS306N	GAL1pr-hMbCas12a::NLS ::3xHA-CYC1t	stable genomic integration in yeast, inducible expression	this work
**pMaM489**	pRS306N	GAL1pr-hLbCas12a::NLS ::3xHA-CYC1t	stable genomic integration in yeast, inducible expression	this work
**pRS416**		shuttle vector and yeast centromeric plasmid conferring uracil prototrophy		Ref. [15]

(Supplementary Table 5 continued)				
**plasmid**	**backbone**	**description**	**purpose**	**source**
**pRS415**		shuttle vector and yeast centromeric plasmid conferring leucine prototrophy		Ref.[15]
**pBeB503**	pRS416	TEF1pr-hFnCas12a::NLS::3xHA-CYC1t	constitutive expression in yeast	this work
**pBeB603**	pRS416	TEF1pr-hLbCas12a::NLS::3xHA-CYC1t	constitutive expression in yeast	this work
**pBeB703**	pRS416	TEF1pr-hAsCas12a::NLS::3xHA-CYC1t	constitutive expression in yeast	this work
**pBeB803**	pRS416	TEF1pr-hMbCas12a::NLS::3xHA-CYC1t	constitutive expression in yeast	this work
**pBeB501**	pRS415	GAL1pr-hFnCas12a::NLS::3xHA-CYC1t	inducible expression in yeast	this work
**pBeB601**	pRS415	GAL1pr-hLbCas12a::NLS::3xHA-CYC1t	inducible expression in yeast	this work
**pBeB701**	pRS415	GAL1pr-hAsCas12a::NLS::3xHA-CYC1t	inducible expression in yeast	this work
**pBeB801**	pRS415	GAL1pr-hMbCas12a::NLS::3xHA-CYC1t	inducible expression in yeast	this work
**pFA6a**		*E. coli* plasmid with ampicillin resistance gene *amp*R		Ref.[16]
**pBeB019**	pFA6a	S3-mScarlet-i-ADH1t (hphNT1) SNR52pr-FnCas12a handle	feature cassette template with a GFP conferring hygromycin resistance	this work
**pBeB013**	pFA6a	S3-mNeonGreen-ADH1t (hphNT1) SNR52pr-FnCas12a handle	feature cassette template with a GFP conferring hygromycin resistance	this work
**pMaM505**	pFA6a	S3-mNeonGreen-ADH1t (kanMX4) SNR52pr-FnCas12a handle	feature cassette template with a GFP conferring G-418 resistance	this work
**pBeB064**	pFA6a	mNeonGreen-ADH1t(kanMX4) SNR52pr-t(GCC)F1-FnCas12a handle	feature cassette template with a GFP conferring G-418 resistance	this work
**pKH51**	pFA6a	mNeonGreen-ADH1t(hphNT1) SNR52pr-t(GCC)F1-FnCas12a handle	feature cassette template with a GFP conferring hygromycin resistance	this work

Supplementary Table 6. Primers used in this study.

primer name	primer sequence (5'-to-3')
**S3-GPD1-BamHI**	CTATGGATCCAAGAACCTGCCGGACATGATTGAAGAATTAGATCTACATGAAGATCGTACGCTGCAGGTCGAC
**GPD1 3'-HA with FnCas12a [crRNA]GPD1+1182**	ATAGGATCCGTGGGGGAAAGTATGATATGTTATCTTTCTCCAATAAATCTAAAAAAAAGATATGTTATCTTTCTCCAAATCTACAACAGTAGAAATTAGATCATTTATCTTTCACTGCGG
**S3-PIL1-BamHI**	CTATGGATCCGTCGGACACCAGCAAAGTGAGTCTCTTCCCCAACAAACAACAGCTCGTACGCTGCAGGTCGAC
**PIL1 3'-HA with FnCas12a [crRNA]PIL1+1022**	ATAGGATCCTTTTTTTTTTGTTTCTAATAGATTGTTGATTTATTTTGATTAAAAAAAACAACAAACAACAGCTTAATCATCTACAACAGTAGAAATTAGATCATTTATCTTTCACTGCGG
**S3-MDH1-BamHI**	CTATGGATCCTTGAAGAAGAATATCGAAAAGGGTGTCAACTTTGTTGCTAGTAAACGTACGCTGCAGGTCGAC
**MDH1 3'-HA with FnCas12a [crRNA]MDH1+998**	ATAGGATCCTTTTCCCTATTTTTCACTCTATTTCTGATCTTGAACAATCTAAAAAAAAGGTGTCAACTTTGTTGCTAGATCTACAACAGTAGAAATTAGATCATTTATCTTTCACTGCGG
**S3-ENO2-BamHI**	CTATGGATCCAAGGCTGTCTACGCCGGTGAAAACTTCCACCACGGTGACAAGTTGCGTACGCTGCAGGTCGAC
**ENO2 3'-HA with FnCas12a [crRNA]ENO2+1311**	ATAGGATCCATAAGCAGAAAAGACTAATAATTCTTAGTTAAAAGCACTTTAAAAAAAATCCACCACGGTGACAAGTTGATCTACAACAGTAGAAATTAGATCATTTATCTTTCACTGCGG
**ENO2 3'-HA with FnCas12a [crRNA]ENO2+1329**	ATAGGATCCATAAGCAGAAAAGACTAATAATTCTTAGTTAAAAGCACTTTAAAAAAAATGTAAAGTGCTTTTAACTAAATCTACAACAGTAGAAATTAGATCATTTATCTTTCACTGCGG
**ENO2 3'-HA with AsCas12a [crRNA]ENO2+1329**	ATAGGATCCATAAGCAGAAAAGACTAATAATTCTTAGTTAAAAGCACTTTAAAAAAAATCCACCACGGTGACAAGTTGATCTACAAGAGTAGAAATTAGATCATTTATCTTTCACTGCGG
**ENO2 3'-HA with MbCas12a [crRNA]ENO2+1329**	ATAGGATCCTAAGCAGAAAAGACTAATAATTCTTAGTTAAAAGCACTTTAAAAAAAATCCACCACGGTGACAAGTTGATCTACAAACAGTAGAAATTTGATCATTTATCTTTCACTGCGG
**ENO2 3'-HA with LbCas12a [crRNA]ENO2+1329**	ATAGGATCCTAAGCAGAAAAGACTAATAATTCTTAGTTAAAAGCACTTTAAAAAAAATCCACCACGGTGACAAGTTGATCTACACTTAGTAGAAATTAGATCATTTATCTTTCACTGCGG
**S3-PDC1-BamHI**	CTATGGATCCTTGGTTGAACAAGCTAAGTTGACTGCTGCTACCAACGCTAAGCAACGTACGCTGCAGGTCGAC
**PDC1 3'-HA with FnCas12a [crRNA]PDC1+1701**	ATAGGATCCGCTTATAAAACTTTAACTAATAATTAGAGATTAAATCGCTTAAAAAAAATTTAACTAATAATTAGAGATATCTACAACAGTAGAAATTAGATCATTTATCTTTCACTGCGG
**PDC1 3'-HA with FnCas12a [crRNA]ADE2+241**	ATAGGATCCGCTTATAAAACTTTAACTAATAATTAGAGATTAAATCGCTTAAAAAAAAGCATGTTGATGTTCCTACACATCTACAACAGTAGAAATTAGATCATTTATCTTTCACTGCGG
**PDC1 3'-HA with FnCas12a [crRNA]PDC1+1705**	ATAGGATCCGCTTATAAAACTTTAACTAATAATTAGAGATTAAATCGCTTAAAAAAAAGCAATAAGCGATTTAATCTCATCTACAACAGTAGAAATTAGATCATTTATCTTTCACTGCGG
**PDC1 3'-HA with AsCas12a [crRNA]PDC1+1701**	ATAGGATCCGCTTATAAAACTTTAACTAATAATTAGAGATTAAATCGCTTAAAAAAAATTTAACTAATAATTAGAGATATCTACAAGAGTAGAAATTAGATCATTTATCTTTCACTGCGG
**PDC1 3'-HA with MbCas12a [crRNA]PDC1+1701**	ATAGGATCCCTTATAAAACTTTAACTAATAATTAGAGATTAAATCGCTTAAAAAAAATTTAACTAATAATTAGAGATATCTACAAACAGTAGAAATTTGATCATTTATCTTTCACTGCGG
**PDC1 3'-HA with LbCas12a [crRNA]PDC1+1701**	ATAGGATCCCTTATAAAACTTTAACTAATAATTAGAGATTAAATCGCTTAAAAAAAATTTAACTAATAATTAGAGATATCTACACTTAGTAGAAATTAGATCATTTATCTTTCACTGCGG

**pool-FP1**	GATAAATGATCTAATTTCTACTGTTGTAG
**pool-RP1**	AGTAGAAGTATTGCCGGC
**pool-FP2**	GGGCTTGCGCATAATTTCTACTGTTGTAGAT
**pool-RP2**	ACGACCGCCTGAAGAGCC
**pool-FP3**	GGGCTTGCGCATAATTTCTACTGTTGTAG
**pool-RP3**	GTGCACCGCCTGAAGAGCC
**cassette-FP1**	GCCGGCAATACTTCTACTCGTACGCTGCAGGTCGAC
**cassette-RP1**	ATCTACAACAGTAGAAATTAGATCATTTATCTTTC
**cassette-FP2 (-FP3)**	GGCTCTTCAGGCGGTGCACGTACGCTGCAGGTCGAC
**cassette-RP2 (-RP3)**	ATCTACAACAGTAGAAATTATGCGCAAGCCC
**vect_bubble-no_BC-Watson**	/PHOS/CGTCCTCTCCTGCTCTGTAGCCTTCTCGTGTGCAGACTTGAGGTGAGTGGCTCTCTTCCCTCT/3DDC/
**vect_bubble-no_BC-Crick**	CCGAGAGGGAAGAGAGCGTGACTGGAGTTCAGACGTGTGCTCTTCCGATCTGAGCAGGAGAGGACGT
**vect_bubble-BC1-Watson**	/PHOS/GAGAAGCAACGTCCTCTCCTGCTCTGTAGCCTTCTCGTGTGCAGACTTGAGGTGAGTGGCTCTCTTCCCTCT/3DDC/
**vect_bubble-BC1-Crick**	CCGAGAGGGAAGAGAGCGTGACTGGAGTTCAGACGTGTGCTCTTCCGATCTGAGCAGGAGAGGACGTTGCTTCTCT
**vect_bubble-BC2-Watson**	/PHOS/CTTACCGCTCGTCCTCTCCTGCTCTGTAGCCTTCTCGTGTGCAGACTTGAGGTGAGTGGCTCTCTTCCCTCT/3DDC/
**vect_bubble-BC2-Crick**	CCGAGAGGGAAGAGAGCGTGACTGGAGTTCAGACGTGTGCTCTTCCGATCTGAGCAGGAGAGGACGAGCGGTAAGT
**vect_bubble-BC3-Watson**	/PHOS/ACTCTCAAGCGTCCTCTCCTGCTCTGTAGCCTTCTCGTGTGCAGACTTGAGGTGAGTGGCTCTCTTCCCTCT/3DDC/
**vect_bubble-BC3-Crick**	CCGAGAGGGAAGAGAGCGTGACTGGAGTTCAGACGTGTGCTCTTCCGATCTGAGCAGGAGAGGACGCTTGAGAGTT
**vect_bubble-BC4-Watson**	/PHOS/CCAACAGAACGTCCTCTCCTGCTCTGTAGCCTTCTCGTGTGCAGACTTGAGGTGAGTGGCTCTCTTCCCTCT/3DDC/
**vect_bubble-BC4-Crick**	CCGAGAGGGAAGAGAGCGTGACTGGAGTTCAGACGTGTGCTCTTCCGATCTGAGCAGGAGAGGACGTTCTGTTGGT
**vect_initiating_primer**	GTTCAGACGTGTGCTCTTCCG
**vect_specific-mNeon-BC1**	ACACTCTTTCCCTACACGACGCTCTTCCGATCTNNNNNNNNCGCTAACTCGCCATGTTGTCTTCTTCACC
**vect_specific-mNeon-BC2**	ACACTCTTTCCCTACACGACGCTCTTCCGATCTNNNNNNNNGAGAGACCACTGCCATGTTGTCTTCTTCACC
**vect_specific-mNeon-BC3**	ACACTCTTTCCCTACACGACGCTCTTCCGATCTNNNNNNNNCCAGAAGAAGCCATGTTGTCTTCTTCACC
**vect_specific-mScarlet-BC1**	ACACTCTTTCCCTACACGACGCTCTTCCGATCTNNNNNNNNAACCTTGTCGCTCCTTGATCACTGCCTCGC
**vect_specific-mScarlet-BC2**	ACACTCTTTCCCTACACGACGCTCTTCCGATCTNNNNNNNNTGTGTCCGTCTCCTTGATCACTGCCTCGC
**vect_specific-SNR52-BC1**	ACACTCTTTCCCTACACGACGCTCTTCCGATCTNNNNNNNNCAATCCGCAAGCAGTGAAAGATAAATGATCTAATTTCTACTG
**vect_specific-SNR52-BC2**	ACACTCTTTCCCTACACGACGCTCTTCCGATCTNNNNNNNNGCGTATTGAGCAGTGAAAGATAAATGATCTAATTTCTACTG
**vect_specific-SNR52-BC3**	ACACTCTTTCCCTACACGACGCTCTTCCGATCTNNNNNNNNGCAACGTTCAGCAGTGAAAGATAAATGATCTAATTTCTACTG

**vect_specific-SNR52-BC4**	ACACTCTTTCCCTACACGACGCTCTTCCGATCTNNNNNNNNATCTGGAGTTAGCAGTGAAAGATAAATGATCTAATTTCTACTG
**vect_illumina-P5**	AATGATACGGCGACCACCGAGATCTACACTCTTTCCCTACACGACGCTC
**vect_illumina-P7**	CAAGCAGAAGACGGCATACGAGATGGCCACTGTGACTGGAGTTCAGACGTGTGCTCTTCCGATC
**halfY-Rd2-Watson**	/PHOS/CGTCCTCTCCTGCTC/SPCC3/
**halfY-Rd2-N8-Crick**	GATCGATCGATCGTTCAGACGTGTGCTCTTCCGATCTNNNNNNNN(TT)GAGCAGGAGAGGACGT
**halfY-Rd2-N8T-Crick**	GATCGATCGATCGTTCAGACGTGTGCTCTTCCGATCTNNNNNNNNTGAGCAGGAGAGGACGT
**halfY-Rd2-N8T2-Crick**	GATCGATCGATCGTTCAGACGTGTGCTCTTCCGATCTNNNNNNNNTTGAGCAGGAGAGGACGT
**Vect_specific-FnCas12a-handle-N6**	ACACTCTTTCCCTACACGACGCTCTTCCGATCTNNNNNNCTTGCGCATAATTTCTACTGTTG
**Vect_specific-FnCas12a-handle-N6G**	ACACTCTTTCCCTACACGACGCTCTTCCGATCTNNNNNNGCTTGCGCATAATTTCTACTGTTG
**Vect_specific-FnCas12a-handle-N6G2**	ACACTCTTTCCCTACACGACGCTCTTCCGATCTNNNNNNGGCTTGCGCATAATTTCTACTGTTG
**tRNA-seq-N6.fw**	ACACTCTTTCCCTACACGACGCTCNNNNNNGTTCGATTCCGGGCTTG
**tRNA-seq-N6T.fw**	ACACTCTTTCCCTACACGACGCTCNNNNNNTGTTCGATTCCGGGCTTG
**tRNA-seq-N6T2.fw**	ACACTCTTTCCCTACACGACGCTCNNNNNNTTGTTCGATTCCGGGCTTG
**mNeon-seq-N6.rev**	GTTCAGACGTGTGCTCTTCCGATCTNNNNNNGTCTTCTTCACCCTTAGAAACC
**mNeon-seq-N6T.rev**	GTTCAGACGTGTGCTCTTCCGATCTNNNNNNTGTCTTCTTCACCCTTAGAAACC
**mNeon-seq-N6T2.rev**	GTTCAGACGTGTGCTCTTCCGATCTNNNNNNTTGTCTTCTTCACCCTTAGAAACC
**P023poolseqN6.fw**	ACACTCTTTCCCTACACGACGCTCNNNNNNGGCTTGCGCATAATTTC
**P023poolseqN6T.fw**	ACACTCTTTCCCTACACGACGCTCNNNNNNTGGCTTGCGCATAATTTC
**P023poolseqN6T2.fw**	ACACTCTTTCCCTACACGACGCTCNNNNNNTTGGCTTGCGCATAATTTC
**P023poolseqN6.rv**	GTTCAGACGTGTGCTCTTCCGATCTNNNNNNGTGCACCGCCTGAAGAG
**P023poolseqN6T.rv**	GTTCAGACGTGTGCTCTTCCGATCTNNNNNNTGTGCACCGCCTGAAGAG
**P023poolseqN6T2.rv**	GTTCAGACGTGTGCTCTTCCGATCTNNNNNNTTGTGCACCGCCTGAAGAG
**III-ONP-P5-bi501**	AATGATACGGCGACCACCGAGATCTACACTGTGGCGCAAGCTTACACTCTTTCCCTACACGACGCTC
**III-ONP-P5-bi502**	AATGATACGGCGACCACCGAGATCTACACCGCGATCTAGGAAGACACTCTTTCCCTACACGACGCTC
**III-ONP-P5-bi503**	AATGATACGGCGACCACCGAGATCTACACAATGACGAGCGAATACACTCTTTCCCTACACGACGCTC
**III-ONP-P5-bi504**	AATGATACGGCGACCACCGAGATCTACACTCAGAACTCTCGAAACACTCTTTCCCTACACGACGCTC
**III-ONP-P5-bi505**	AATGATACGGCGACCACCGAGATCTACACTTCCTACGAACAGAACACTCTTTCCCTACACGACGCTC
**III-ONP-P5-bi506**	AATGATACGGCGACCACCGAGATCTACACATTCGGTTGCCTAGACACTCTTTCCCTACACGACGCTC
**III-ONP-P5-bi507**	AATGATACGGCGACCACCGAGATCTACACCACATAACAGTTGGACACTCTTTCCCTACACGACGCTC
**III-ONP-P5-bi508**	AATGATACGGCGACCACCGAGATCTACACGTGGATTCTCCAGTACACTCTTTCCCTACACGACGCTC
**III-ONP-P5-bi509**	AATGATACGGCGACCACCGAGATCTACACACCTTCTCGATGGAACACTCTTTCCCTACACGACGCTC

**III-ONP-P5-bi510**	AATGATACGGCGACCACCGAGATCTACACCTCTCCTTCCGGTTACACTCTTTCCCTACACGACGCTC
**III-ONP-P7-bi701**	CAAGCAGAAGACGGCATACGAGATTGTGGCGCAAGCTTGTGACTGGAGTTCAGACGTGTGCTCTTCCGATC
**III-ONP-P7-bi702**	CAAGCAGAAGACGGCATACGAGATCGCGATCTAGGAAGGTGACTGGAGTTCAGACGTGTGCTCTTCCGATC
**III-ONP-P7-bi703**	CAAGCAGAAGACGGCATACGAGATAATGACGAGCGAATGTGACTGGAGTTCAGACGTGTGCTCTTCCGATC
**III-ONP-P7-bi704**	CAAGCAGAAGACGGCATACGAGATTCAGAACTCTCGAAGTGACTGGAGTTCAGACGTGTGCTCTTCCGATC
**III-ONP-P7-bi705**	CAAGCAGAAGACGGCATACGAGATTTCCTACGAACAGAGTGACTGGAGTTCAGACGTGTGCTCTTCCGATC
**III-ONP-P7-bi706**	CAAGCAGAAGACGGCATACGAGATATTCGGTTGCCTAGGTGACTGGAGTTCAGACGTGTGCTCTTCCGATC
**III-ONP-P7-bi707**	CAAGCAGAAGACGGCATACGAGATCACATAACAGTTGGGTGACTGGAGTTCAGACGTGTGCTCTTCCGATC
**III-ONP-P7-bi708**	CAAGCAGAAGACGGCATACGAGATGTGGATTCTCCAGTGTGACTGGAGTTCAGACGTGTGCTCTTCCGATC
**III-ONP-P7-bi709**	CAAGCAGAAGACGGCATACGAGATACCTTCTCGATGGAGTGACTGGAGTTCAGACGTGTGCTCTTCCGATC
**III-ONP-P7-bi710**	CAAGCAGAAGACGGCATACGAGATCTCTCCTTCCGGTTGTGACTGGAGTTCAGACGTGTGCTCTTCCGATC
**NegCells-Crick**	AATGATACGGCGACCACCGAGATCTACACNNNNNNNNNNNNNNNTCTTGCCAGATCGTCGGCAGCGTCAGATGTGTATAAGAGACAG
**NegCells-Watson**	/PHOS/CTGTCTCTTATACACATCT/SPCC3/
**NegCells-UP-1stPCR**	CCGAAGATGTGCAATTCGTGAGTAGC
**NegCells-DOWN-1stPCR**	GACGCTCGAAGGCTTTAACGAGC
**NegCells-1st_2ndPCR**	AATGATACGGCGACCACCGAGATC
**NegCells-UP-1ndPCR**	CAAGCAGAAGACGGCATACGAGATGCAAGCTTGTCTCGTGGGCTCGGAGATGTGTATAAGAGACAGAAGAAGCCATGTTGTCTTCTTCACC
**NegCells-DOWN-1ndPCR**	CAAGCAGAAGACGGCATACGAGATCTAGGAAGGTCTCGTGGGCTCGGAGATGTGTATAAGAGACAGGAAAGATAAATGATCGCGCAAGTG

Supplementary Table 7. Pool designs.

Oligo-pool ID	PAM space	degeneracy		oligo-nucleotide length	supplier
		sequences	ORFs		
**pool A**					CustomArray Inc.
**pool B1**	TTV (1,577)	1,577	215 nuclear ORFs	160 nt	Twist Bioscience
**pool B2**					Twist Bioscience
**pool C**	TTV (11,155) +TYN (1,317)	12,472	5,664	170 nt	CustomArray Inc.
**pool D**	TTV (27,000)	27,000	5,940	160 nt	Agilent Technologies

Supplementary Data

Supplementary Data 1. 1,577 oligonucleotide pool sequences for the small library targeting 215 nuclear proteins with nuclear localization.

Data provided as Online Supplementary Material.

Supplementary Data 2. Oligonucleotide pool sequences for the first genome-wide library by ORF. Some of the unique set of 12,472 sequences can target more than a single ORF, which is why a total number of 12,514 entries is provided. We excluded seven entries for *YEL020W-A*, *YEL020C-B*, *YEL021W*, and *YEL022W*, which are near to *ura3*-52, the locus at which the Cas12a-family proteins were integrated in this study (Supplementary Note 2).

Data provided as Online Supplementary Material.

Supplementary Table 3. Oligonucleotide pool sequences for the second genome-wide library by ORF. Some of the unique set of 27,000 sequences can target more than a single ORF, which is why a total number of 27,640 entries is provided. We excluded 15 entries for *YEL020W-A*, *YEL020C-B*, *YEL021W*, and *YEL022W*, which are near to *ura3*-52, the locus at which the Cas12a-family proteins were integrated in this study (Supplementary Note 2).

Data provided as Online Supplementary Material.

SUPPLEMENTARY REFERENCES

1. Engel, S. R. *et al.* The reference genome sequence of *Saccharomyces cerevisiae:* then and now. G3 **4,** 389-398 (2014).
2. Zetsche, B. *et al.* Cpf1 Is a Single RNA-Guided Endonuclease of a Class 2 CRISPR-Cas System. *Cell* **163,** 759-771 (2015).
3. Hammond, A. *et al.* A CRISPR-Cas9 gene drive system targeting female reproduction in the malaria mosquito vector *Anopheles gambiae*. *Nat Biotechnol* **34,** 78-83 (2016).
4. Sinkins, S. P. & Gould, F. Gene drive systems for insect disease vectors. *Nat Rev Genet* **7,** 427-435 (2006).
5. Committee on Gene Drive Research in Non-Human Organisms: Recommendations for Responsible Conduct, Board on Life Sciences, Division on Earth and Life Studies. National Academies of Sciences, Engineering, and Medicine. in *Advancing Science, Navigating Uncertainty, and Aligning Research with Public Values* (National Academies Press (US), 2016). doi:10.17226/23405
6. Esvelt, K. M., Smidler, A. L., Catteruccia, F. & Church, G. M. Concerning RNA-guided gene drives for the alteration of wild populations. *eLife* **3,** (2014).
7. Zhang, L., Kasif, S., Cantor, C. R. & Broude, N. E. GC/AT-content spikes as genomic punctuation marks. *Proc Natl Acad Sci U.S.A.* **101,** 16855-16860 (2004).
8. Kaplan, N. *et al.* The DNA-encoded nucleosome organization of a eukaryotic genome. *Nature* **458,** 362-366 (2009).
9. Jordán-Pla, A. *et al.* Chromatin-dependent regulation of RNA polymerases II and III activity throughout the transcription cycle. *Nucleic Acids Res* **43,** 787-802 (2015).
10. Ho, B., Baryshnikova, A. & Brown, G. W. Unification of Protein Abundance Datasets Yields a Quantitative *Saccharomyces cerevisiae* Proteome. *Cell Syst* **6,** 192-205.e3 (2018).
11. Meurer, M. *et al.* Genome-wide C-SWAT library for high-throughput yeast genome tagging. *Nat Methods* **425,** 1 (2018).
12. Wang, M. *et al.* PaxDb, a database of protein abundance averages across all three domains of life. *Mol Cell Proteomics* **11,** 492-500 (2012).
13. Knop, M. & Schiebel, E. Receptors determine the cellular localization of a gamma-tubulin complex and thereby the site of microtubule formation. *EMBO J* **17,** 3952-3967 (1998).
14. Taxis, C. & Knop, M. System of centromeric, episomal, and integrative vectors based on drug resistance markers for *Saccharomyces cerevisiae*. *BioTechniques* **40,** 73-78 (2006).

15. Sikorski, R. S. & Hieter, P. A system of shuttle vectors and yeast host strains designed for efficient manipulation of DNA in *Saccharomyces cerevisiae*. *Genetics* **122,** 19-27 (1989).
16. Wach, A., Brachat, A., Pöhlmann, R. & Philippsen, P. New heterologous modules for classical or PCR-based gene disruptions in *Saccharomyces cerevisiae*. *Yeast* **10,** 1793-1808 (1994).
