# Supplementary material for: Pooled clone collections by multiplexed CRISPR-Cas12a-assisted gene tagging in yeast
Source: Nat Commun. 2019 Jul 4;10:2960. doi: 10.1038/s41467-019-10816-7 (PMC6609715; doi:10.1038/s41467-019-10816-7)
Supplement: Supplementary file 5 — Description of Additional Supplementary Files [file 41467_2019_10816_MOESM5_ESM.docx]

**Table: Supplementary Data 1
Description:** 1,577 oligonucleotide pool sequences for the small library targeting 215 nuclear proteins with nuclear localization.

**Table:**  **Supplementary Data 2**
**Description:** Oligonucleotide pool sequences for the first genome-wide library by ORF. Some of the unique set of 12,472 sequences can target more than a single ORF, which is why a total number of 12,514 entries is provided. We excluded seven entries for YEL020W-A, YEL020C-B, YEL021W, and YEL022W, which are near to ura3-52, the locus at which the Cas12a-family proteins were integrated in this study (Supplementary Note 2).

**Table: Supplementary data 3**
**Description:** Oligonucleotide pool sequences for the second genome-wide library by ORF. Some of the unique set of 27,000 sequences can target more than a single ORF, which is why a total number of 27,640 entries is provided. We excluded 15 entries for YEL020W-A, YEL020C-B, YEL021W, and YEL022W, which are near to ura3-52, the locus at which the Cas12a-family proteins were integrated in this study (Supplementary Note 2)
