# Supplementary material for: Pooled clone collections by multiplexed CRISPR-Cas12a-assisted gene tagging in yeast
Source: Nat Commun. 2019 Jul 4;10:2960. doi: 10.1038/s41467-019-10816-7 (PMC6609715; doi:10.1038/s41467-019-10816-7)
Supplement: Supplementary file 7 — Source Data [file 41467_2019_10816_MOESM7_ESM.zip › Source_Data-Description.docx]

**SOURCE DATA**

**for**

Buchmuller and Herbst *et al.*

**Pooled clone collections by multiplexed
CRISPR-Cas12a-assisted gene tagging in yeast**

**CONTENTS**

**Figure 1b.** Table with raw counts is provided.

**Figure 1c.** Original high-resolution images of plates and original uncropped fluorescence microscopy images are provided as stacks.

**Figure 1d.** Table with counts is provided.

**Figure 3b, 3c, 3e.** Table with number of occurrences is provided. Note: Column “certain” indicates whether the respective entity is a member of the lower 25% by copy number (light gray in Fig. 3b–c).

**Figure 3d.** Table with read counts after thresholding is provided.

**Figure 3f.** Table with read counts after thresholding is provided.

**Figure 3g.** Original high-resolution fluorescence microscopy and bright-field images are provided.

**Figure 3h.** Table with number of occurrences is provided.

**Figure 3i.** Table with number of occurrences is provided.

**Figure 4a.** Table with thresholded/down-sampled read counts is provided. The file also contains information about the reference it was aligned to and its CIGAR string.

**Figure 4b.** The original high-resolution microscopy images are provided as stack with bright-field, short and long exposure time. Examples “cytosol”, “mitochondria”, and “cell periphery” are crops from Well H08, “nucleus”, and “nuclear periphery” from Well H10.

**Figure 4c.** Table with read counts after thresholding is provided.

**Figure 4d.** See Supplementary Fig. 8.

**Figure 5b, 5c.** Table with UMI counts by library, tagged ORF and the tagging oligonucleotide is provided. Note that the figure summarizes UMI counts by ORF.

**Figure 5d.** The recorded FACS data is provided.

**Figure 5e.** The source code and data to create this graph with R is provided.

**Figure 5f.** The source code and data to create this graph with R is provided.

**Figure 5g.** The original high-resolution microscopy images are provided as stack.

**Supplementary Fig. 1.** The source code and data to create this graph with R is provided.

**Supplementary Fig. 2.** Original high-resolution images are provided.

**Supplementary Fig. 3a.** Original high-resolution images are provided as stack. The original file to quantitate clone numbers is provided.

**Supplementary Fig. 3b.** Original high-resolution images are provided.

**Supplementary Fig. 3c.** Original full blot is provided in high-resolution. In addition, the bright-field image showing the marker (BioRad Precision Plus Protein Dual Color Standard) is provided.


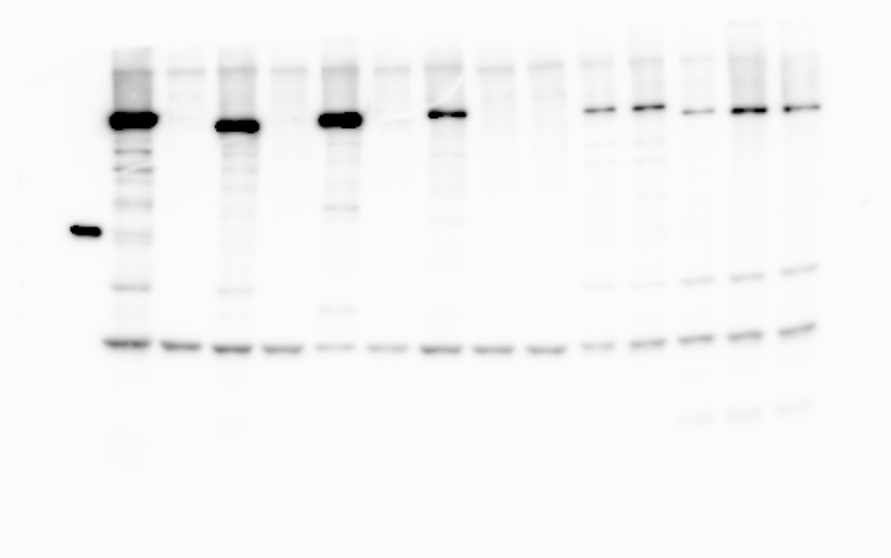


1 2 3 4 5 6 7 8 9 10 11 12 13 14 15

1: Marker (BioRad Precision Plus Protein Dual Color Standard)

2: YBeB500 (FnCas12a-NLS-3xHA) in YP + 2% (w/v) galactose + 2% (w/v) raffinose

3: YBeB500 (FnCas12a-NLS-3xHA) in YPD

4: YBeB600 (LbCas12a-NLS-3xHA) in YP + 2% (w/v) galactose + 2% (w/v) raffinose

5: YBeB600 (LbCas12a-NLS-3xHA) in YPD

6: YBeB700 (AsCas12a-NLS-3xHA) in YP + 2% (w/v) galactose + 2% (w/v) raffinose

7: YBeB700 (AsCas12a-NLS-3xHA) in YPD

8: YBeB800 (MbCas12a-NLS-3xHA) in YP + 2% (w/v) galactose + 2% (w/v) raffinose

9: YBeB800 (MbCas12a-NLS-3xHA) in YPD

10–15: not shown

**Supplementary Fig. 4.** The source code and data to create this graph with R is provided.

**Supplementary Fig. 5a.** Original full gel is provided in high-resolution. The cropped image shows lanes 1–5 thereof.

**Supplementary Fig. 5b.** Original full gel is provided in high-resolution. The cropped image shows lanes 1–3 thereof.

**Supplementary Fig. 5c.** Original full gel is provided in high-resolution. The cropped image shows lanes 1–3 thereof.

**Supplementary Fig. 5d.** Original full gel is provided in high-resolution. The cropped image shows lanes 1–3 thereof.

**Supplementary Fig. 8b.** The source code and data to create this graph with R is provided.

**Supplementary Fig. 9a.** The source code and data to create this graph with R is provided.

**Supplementary Fig. 10.** The source code and data to create this graph with R is provided.

**Supplementary Table 3.** The sequencing report (MinION) and the processed read counts thereof are provided.
