# Supplementary material for: Pooled clone collections by multiplexed CRISPR-Cas12a-assisted gene tagging in yeast
Source: Nat Commun. 2019 Jul 4;10:2960. doi: 10.1038/s41467-019-10816-7 (PMC6609715; doi:10.1038/s41467-019-10816-7)
Supplement: Supplementary file 7 — Source Data [file 41467_2019_10816_MOESM7_ESM.zip › Source_Data-Sup_Table_3.zip!/Sup_Table_3/T03-MinION_sequencing_report.pdf]

MinKNOW

Protocol
NC_6Hr_control_FLO-MIN106_SQK-RNA001_plus_Basecaller
MinION ID
MN21357
ASIC ID
4245857692
Sample ID
mNeon_lib
Flowcell ID
FAH25882
Host
tuananh-desktop
MinKNOW Version
1.7.14
Protocols Version
1.7.14

Read Summary

Total 940722 **Events** 300794694 **Channels** 501 of 512

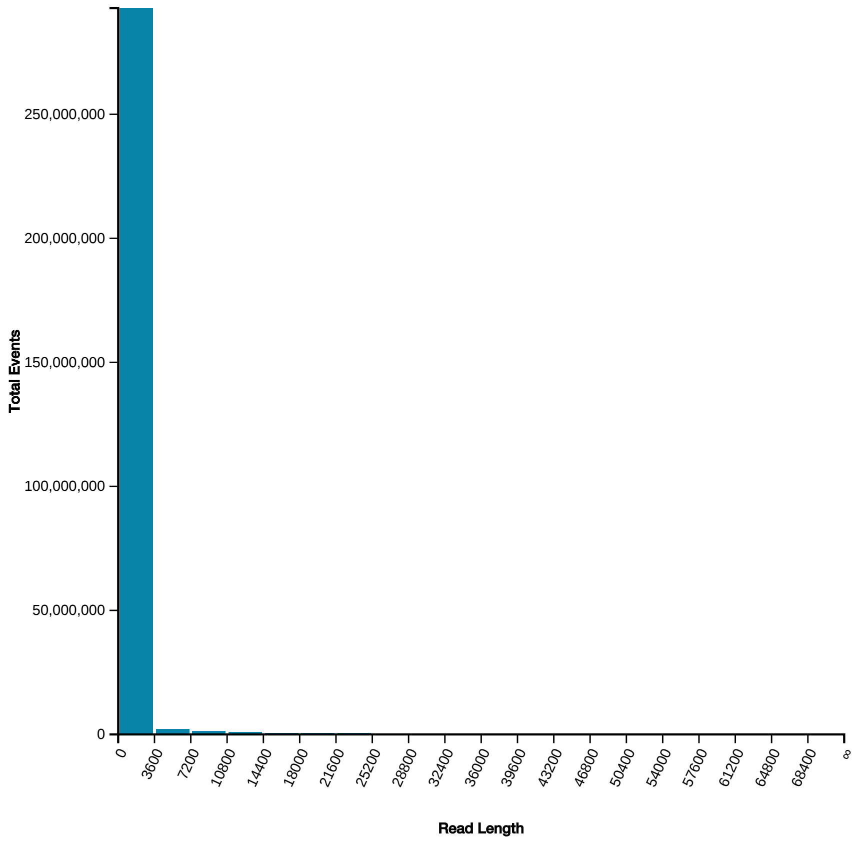

CSV PDF

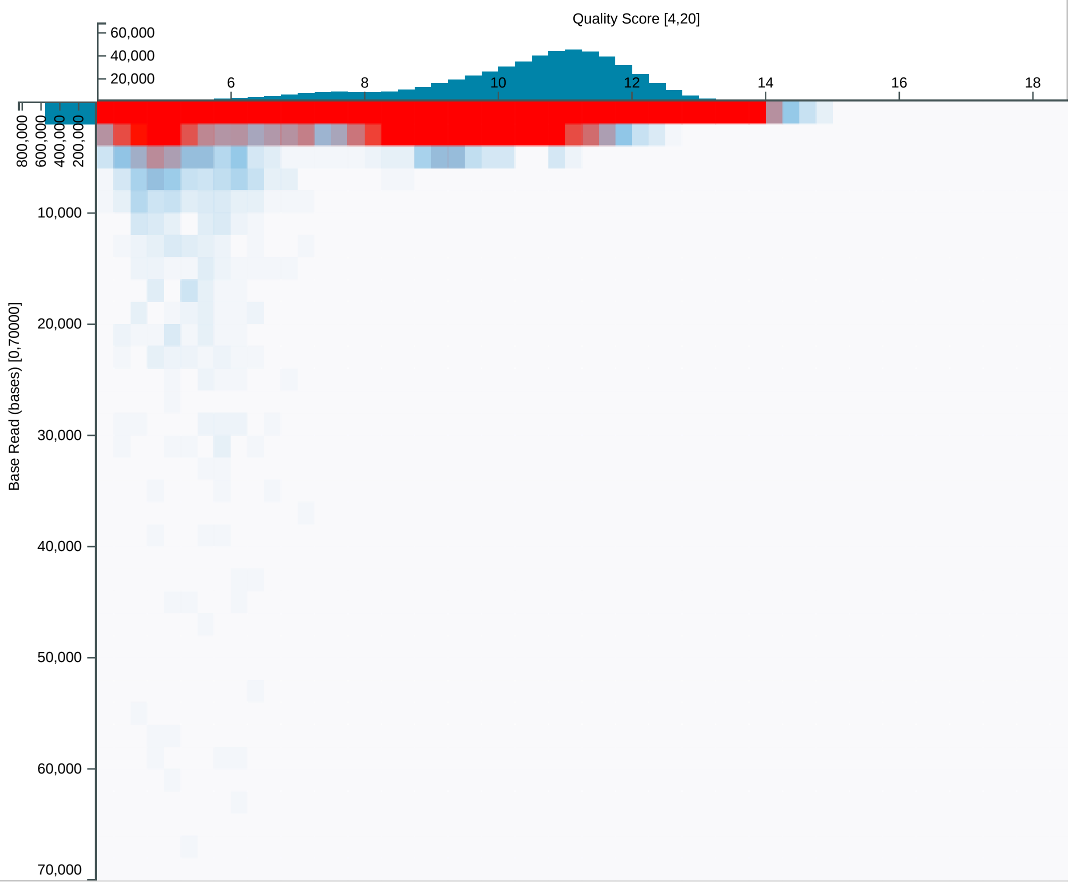

© 2008 - 2016 Oxford Nanopore Technologies
